# Unusual Finding in an Unintentional, Non-fire-Related Carbon Monoxide Poisoning Death: Subarachnoidal Haemorrhage

**DOI:** 10.5152/eurasianjmed.2021.20287

**Published:** 2021-10

**Authors:** Yiğit Sezer, Elif Kesmen Öz, Ahmet Şahpaz, Mustafa Talip Şener, Ahmet Nezih Kök

**Affiliations:** 1 Morgue Department, Council of Forensic Medicine, Erzurum Branch, Erzurum, Turkey; 2 Department of Toxicology, Council of Forensic Medicine, Erzurum Branch, Erzurum, Turkey; 3 Department of Forensic Medicine, Atatürk University School of Medicine, Erzurum, Turkey

## Dear Editor,

Carbon monoxide (CO) is the cause of a significant percentage of fatal poisonings in many countries. Subarachnoid haemorrhages are also an important public health issue all over the world. Between 1999 and 2010, there were 5149 reported deaths from unintentional, non-fire-related CO poisoning in the United States.^1^ Between 2008 and 2017, 2667 deaths from CO poisoning were reported in Turkey.^2^

**Figure 1.**
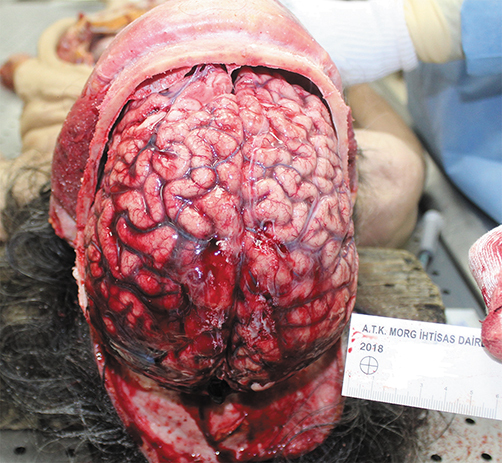
Subarachnoid haemorrhage.

In this case, nonaneurysmatic subarachnoid haemorrhage with a moderate-level CO poisoning death case is presented to mention this rare coincidence in the point of mechanism of death. A 55-year-old woman living alone with no significant past medical history was found dead at her home. She was dressed casually and laid down on the floor. There was an extinguished stove near her in the same room. According to crime scene investigation, there were strong soot and smoke smell in the room where she was found dead. On external examination, her livor mortis was found to be slightly cherry red. There was no traumatic lesion detected, including the scalp, temporal muscles in her head and other parts of the body. There was a cherry-red coloration in the organs and serosal surfaces. Cerebrum and cerebellum were hyperemic, and there was no aneurysmatic change in the vertebrobasilar and other parts of cerebral arteries. There was a 8 × 6 cm^2^ subarachnoid haemorrhage in the bilateral parietal region (Figure 1). On histopathological examination, subarachnoid haemorrhage; parenchymal congestion in brain, perivascular and interstitial fibrosis in the myocardium; mild to moderate stenosis findings in the left circumflex artery, right coronary artery and left ascending coronary artery were detected. Oedema in lungs and congestion in the liver and kidney were detected. COHb (37.6%) was detected in the heart blood with CO oximetry, and no other substances were detected in blood by using Gas chromatography-mass spectormetry (GC-MS) and Liquid chromatography– tandem mass spectrometry (LC-MS/MS).

Over 50% saturation of CO generally indicates the primary cause of death as CO intoxication.^3^ However, there is not always a correlation between COHb level and morbidity and mortality. Death with initial 3% COHb measurement and a survival with 72.3% COHb levels were reported.^4^ Autopsy revealed no other trauma related to body and head. There was no aneurysm detected as the cause of subarachnoid haemorrhage. She had no known past medical history. The most common cause of subarachnoid haemorrhage is ruptured aneurysms. Cerebral arteriovenous malformations, cerebral venous thrombosis, coagulopathies, tumours and drugs are important but rare cause of subarachnoid haemorrhage. Even in some cases, there is no known cause detected. Case fatality of subarachnoid haemorrhages is also high.^5^

It is difficult to distinguish whether she had hypertensive subarachnoid haemorrhage and got intoxicated to death with the increasing COHb blood level (up to 37.6%) or whether she got intoxicated first and, consequently, the intoxication caused subarachnoid haemorrhage. In this moderate-level CO poisoning case, the contribution of subarachnoid haemorrhage in the mechanism of death cannot be ignored. Determination of relative prognostic weights of different contributing factors was difficult in this case. In case of a fatal CO intoxication with relatively lower blood level of COHb, subarachnoid haemorrhage may be an important contributing factor like cardiac and lung disease, being child or elderly.

**Main Points**

- 37.6% COHb level may not cause death all the time.
- Nonaneurysmal subarachnoid haemorrhages have lower mortality and morbidity rates.
- Rare coincidence of moderate-level (37.6%) CO intoxication and subarachnoid haemorrhage both contributed in the mechanism of death in this case.
